# In Silico and Transcription Analysis of Trehalose-6-phosphate Phosphatase Gene Family of Wheat: Trehalose Synthesis Genes Contribute to Salinity, Drought Stress and Leaf Senescence

**DOI:** 10.3390/genes12111652

**Published:** 2021-10-20

**Authors:** Md Ashraful Islam, Md Mustafizur Rahman, Md Mizanor Rahman, Xiujuan Jin, Lili Sun, Kai Zhao, Shuguang Wang, Ashim Sikdar, Hafeez Noor, Jong-Seong Jeon, Wenjun Zhang, Daizhen Sun

**Affiliations:** 1 State Key Laboratory of Sustainable Dryland Agriculture, College of Agronomy, Shanxi Agricultural University, Taigu 030801, China; a.islam160@nwafu.edu.cn (M.A.I.); 15582408175@163.com (X.J.); 18735430724@163.com (L.S.); zk51712@163.com (K.Z.); wsg6162@126.com (S.W.); hafeeznoorbaloch@gmail.com (H.N.); zhangwenjun9876@163.com (W.Z.); 2 Graduate School of Biotechnology and Crop Biotech Institute, Kyung Hee University, Yongin 17104, Korea; mr10bau2@khu.ac.kr (M.M.R.); mizanor@khu.ac.kr (M.M.R.); jjeon@khu.ac.kr (J.-S.J.); 3 Department of Agroforestry and Environmental Science, Sylhet Agricultural University, Sylhet 3100, Bangladesh; ashim.aes@sau.ac.bd

**Keywords:** in silico, *Cis*-regulatory elements, gene transcription, trehalose-6-phosphate phosphatase, wheat

## Abstract

Trehalose-6-phosphate phosphatase (*TPP*) genes take part in trehalose metabolism and also in stress tolerance, which has been well documented in many species but poorly understood in wheat. The present research has identified a family of 31 *TPP* genes in *Triticum aestivum* L. through homology searches and classified them into five clades by phylogenetic tree analysis, providing evidence of an evolutionary status with *Hordeum vulgare, Brachypodium distachyon* and *Oryza sativa.* The exon-intron distribution revealed a discrete evolutionary history and projected possible gene duplication occurrences. Furthermore, different computational approaches were used to analyze the physical and chemical properties, conserved domains and motifs, subcellular and chromosomal localization, and three-dimensional (3-D) protein structures. *Cis*-regulatory elements (CREs) analysis predicted that *TaTPP* promoters consist of CREs related to plant growth and development, hormones, and stress. Transcriptional analysis revealed that the transcription levels of *TaTPPs* were variable in different developmental stages and organs. In addition, qRT-PCR analysis showed that different *TaTPPs* were induced under salt and drought stresses and during leaf senescence. Therefore, the findings of the present study give fundamental genomic information and possible biological functions of the *TaTPP* gene family in wheat and will provide the path for a better understanding of *TaTPPs* involvement in wheat developmental processes, stress tolerance, and leaf senescence.

## 1. Introduction

Cereals are indeed the single most significant part of the diet for the majority of the global population, with about 60% to 80% of carbohydrates coming straightly from them in developing and under-developing nations, respectively [1]. According to the FAO’s most current predictions, global grain production in 2021 will increase by 1.7% over 2020, achieving 2817 million tons [2]. Wheat (*Triticum aestivum* L.) is the world’s largest extensively grown cereal crop and is among the most often eaten cereals by the world population [3]. The major abiotic stresses that decrease wheat productivity throughout the growing period include water shortages, high temperatures, and salinity [4]. Among them, salinity is a major barrier to crop production, especially in wheat, resulting in a yield loss of 65% in moderately saline soils, by influencing nearly every stage of plant growth and development, including germination, vegetative growth, and reproductive growth [5,6]. This abiotic stress condition results in a decrease in yield related traits that directly affect the yield of cereal crops. Thus, one of the most significant tasks for plant breeders right now is to uncover the genes associated with abiotic stress responses and to cultivate genetically engineered varieties with improved stress tolerance [7,8].

**Figure 1:**
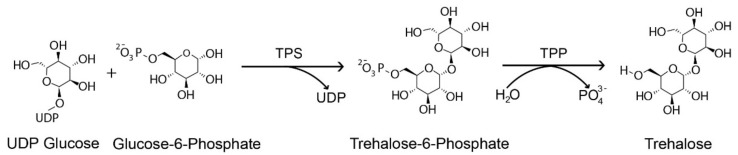
Trehalose biosynthesis pathway in plants. Uridine diphosphate glucose (UDP), Trehalose-6-phosphate synthase (TPS) and Trehalose-6-phosphate phosphatase (TPP).

Plants generate various organic molecules, such as soluble sugar and free amino acids, in response to stress exposure. Trehalose is one of these non-reducing disaccharides composed of two molecules of α-glucose that may accumulate in the cell up to 12% of its dry mass to maintain its integrity and is associated with plant abiotic stress tolerance, including high and low temperature, drought, and osmotic stress tolerance [9,10,11,12]. Many species, including yeast, fungus, invertebrates, plants, bacteria, insects, green weed, and cyanobacteria synthesize this sugar substance [12,13,14,15]. Except for vertebrates, the synthesis of trehalose in plants and other organisms involves two phases with two catalytic enzymes, trehalose-6-phosphate synthase (TPS) and trehalose-6-phosphate phosphatase (TPP). TPS produces trehalose-6-phosphate (T6P), a phosphorylated intermediate, from Uridine diphosphate-glucose (UDPG) and Glucose-6-phosphate (G6P) in the first phase, and the TPP dephosphorylates T6P to produce trehalose in the second phase (Figure 1). Trehalose is then hydrolyzed by an enzyme called trehalase (TRE) to synthesize two molecules of glucose, which suggests that TPS, TPP, and TRE are the three enzymes involved in the trehalose biosynthesis pathway [16]. The *TPS* and *TPP* families encode multiple genes, but *TRE* is denoted by a single copy of the gene [17,18,19].

In addition to providing a route for the production of trehalose, *TPS* and *TPP* have been shown to serve as signaling molecules in higher plants by modulating a variety of plant metabolic and developmental processes. T6P is a signaling metabolite in plants that links growth and development to carbon metabolism and serves as a signal of sucrose status at various phases of the plant’s development [20,21,22]. *TPS* genes were discovered to be involved in the germination of seeds, stress signaling, vegetative phase separation, shoot branching, and flowering time regulation in *Arabidopsis* and rice, with *TPS1* being the most studied [23,24,25,26,27]. Instead, TPP was found to inhibit SnRK1 (Sn1-related protein kinase) activity, a well-known transcriptional regulatory pathway under stress and energy metabolism [28]. The Ramosa1 (RA1) transcription factor activates the transcription of *TPP* to regulate flower branching, which suggests that trehalose may have a role in specific developmental processes [29]. Tobacco plant overexpressing *Escherichia coli TPS* gene *ostA* improved photosynthesis efficiency by enhancing RUBISCO concentration, although *ostB*, a *TPP* gene, exhibited the opposite impact, further suggesting the significance of trehalose in plant photosynthesis [30].

Various studies have reported trehalose enzymes to enhance abiotic and biotic stress tolerance, such as in *Arabidopsis* [31,32]. For example, *ZxTPP* (*Zygophyllum xanthoxylum*) or *ostA* and *ostB* containing tobacco transgenic plants were significantly tolerant to drought [33,34]. Likewise, *ostA* and *ostB* transformed rice plants showed increased trehalose levels and enhanced performance against cold, salt, and drought stresses [35]. Exogenous trehalose triggered a signal transduction pathway including calcium and reactive oxygen species (ROS) and *OsTPP1* or *OsTPP3* transgenic rice and maize plants induced stress-related genes that conferred drought tolerance [36,37,38]. After drought stress, vulnerable maize seedlings had lower *ZmTPP1* expression, whereas resistant seedlings had higher expression [39]. *TPP* promoters’ *Cis*-regulatory elements (CREs) stimulate trehalose metabolism and improve stress response. In *Arabidopsis*, *ABF1*, *ABF2*, and *ABF4* are ABA-responsive elements that directly influence *AtTPPI* expression to increase drought tolerance by changing stomatal apertures [40]. The transcription factor that responds to ABA in the presence of ABA, ABF2 binds directly to the *AtTPPE* promoter, triggering its expression for root elongation and stomatal movement via producing ROS [41]. DREB1A, which binds to the DRE/CR motif in the *AtTPPF* promoter, is thought to upregulate *AtTPPF* transcription in drought-stressed plants [32]. T6P role as a signal for increased carbon availability might have implications for leaf senescence control, as the accumulation of sugars has been demonstrated during leaf senescence in *Arabidopsis*, wheat, tobacco, and maize. The phenotype of mature *otsB*-overexpressing *Arabidopsis* plants included delayed senescence and decreased anthocyanin accumulation, suggesting that the role of *TPP* may perform a crucial role during leaf senescence in plants [42,43,44,45]. To date, *TaTPP-6AL1* and its functional marker have been shown to improve crop yield in wheat [46]. However, the gene structure and regulatory mechanism of wheat *TPPs* are not well studied.

The present study intends to investigate wheat *TPPs* in silico by identification of *TaTPPs,* gene duplication analysis, phylogenetic relationship with other species, subcellular localization prediction, motif and domain analyses, proteins 3-D structure modeling, investigation of CREs, and gene transcription analysis that have all been performed to better understand *TaTPPs* functions in wheat.

## 2. Materials and Methods

### 2.1. Identification of Putative TPPs in the Wheat Genome

To find putative *TPPs* in wheat, we utilized *TPPs* from *Arabidopsis* and rice. Ensembl Plants database was used to collect TPP protein sequences from *Arabidopsis* and rice and a BLASTp search was conducted against the most recent wheat assembly from the IWGSC (RefSeq v1.0) (http://plants.ensembl.org/index.html, 10^−5^ cut-off e-value and bit-score > 100, accessed on 12 March 2021). After eliminating duplicated sequences, SMART (http://smart.embl-heidelberg.de/, accessed on 12 March 2021) or InterPro (https://www.ebi.ac.uk/interpro, accessed on 12 March 2021) and NCBI CDD (https://www.ncbi.nlm.nih.gov/Structure/cdd/wrpsb.cgi, accessed on 12 March 2021) were used to examine the remaining sequences for the presence of transmembrane domains. TPP-related domain-containing protein sequences were collected and designated consecutively according to their chromosomal locations after the sequences without transmembrane domains were deleted. The ProtParam software (https://web.expasy.org/protparam/, accessed on 13 March 2021) was used to calculate the length, molecular weight, isoelectric point (pI), and grand average of hydropathicity (GRAVY) of TPP proteins.

### 2.2. Chromosome Localization, Gene Duplication and Synteny Analysis

*TPPs* genomic locations were acquired from the Ensembl Plants BioMart (http://plants.ensembl.org/biomart/martview, accessed on 14 March 2021) for chromosomal distribution. The *TPPs* were given a ‘Ta’ prefix and were numbered in ascending order according to their ascending chromosomal location. The *TaTPPs* on the wheat chromosomes were represented using TBtools. A NCBI BlastP search (https://blast.ncbi.nlm.nih.gov/Blast.cgi?PROGRAM=blastp&PAGE_TYPE=BlastSearch&BLAST_SPEC=&LINK_LOC=blasttab&LAST_PAGE=blastn, query conditions: percent identity between 75 and 100 and query coverage between 80 and 100, accessed on 14 March 2021) based on the proportion of query cover to the identity of the *TaTPPs* against each other was performed to check for gene duplication [47]. Based on a BLAST search and a phylogenetic tree, duplicate gene pairs were identified. TBtools was used to determine the non-synonymous substitution rate (Ka), synonymous substitution rate (Ks), and Ka/Ks ratio [48]. The synteny relationships of wheat *TPP* genes with different plant species were analyzed using TBtools.

### 2.3. Phylogenetic Analysis, Exon-Intron Distribution and 3-D Structure Modeling

ClustalW in MEGA X was used to align full-length protein sequences from various species [49]. Following the alignment, MEGA X was used to create a phylogenetic tree with the Maximum Likelihood method [50] and 1000 bootstrap values [51]. To examine the exon-intron distribution of *TaTPPs*, the TBtool was used to align the CDSs and genomic sequences. SWISS-MODEL Workspace web tools (https://swissmodel.expasy.org/interactive#sequence, accessed on 16 March 2021), GASS and SOPMA secondary structural method (https://npsa-prabi.ibcp.fr/cgi-bin/npsa_automat.pl?page=npsa%20_sopma.html, accessed on 16 March 2021) and MolProbity server (http://molprobity.biochem.duke.edu/, accessed on 16 March 2021) were used to conduct 3-D structure analyses of TaTPP proteins [52,53,54,55,56,57].

### 2.4. Subcellular Localization Prediction and Protein Domain Analysis

PredSL (http://aias.biol.uoa.gr/PredSL/index.html, accessed on 17 March 2021) was used to predict subcellular localizations. The TPP domain (trehalose-phosphatase (Trehalose PPase); PF02358) was retrieved from the Pfam database and the structures were created with TBtools [58,59]. We utilized MEME suite 5.1.1 to examine TaTPP motifs and The site distribution was set to any number of repetitions, the maximum number of motifs to locate was set to 9, the minimum width was set to 6, the maximum width was set to 50, and the maximum number of motifs to locate was set to 9 [60].

### 2.5. Analysis of Publicly Accessible Expression Data and Cis-Regulatory Elements (CREs)

We used the NCBI database (https://www.ncbi.nlm.nih.gov/, accessed on 19 March 2021) to obtain 2 kb upstream from start codon promoter sequences of 11 *TaTPPs*, which we subsequently submitted to PlantCARE to find the CREs [61]. Netbeans IDE 8.0 (https://netbeans.org., accessed on 25 March 2021) was used to organize data [62] and subsequently TBtools Heatmap was used for data visualization. The Genevestigator RNAseq public anatomy was used to examine gene expression [63] and the MeV tool was then used to visualize expression [64].

### 2.6. Plant Materials and Treatments

*T. aestivum* L. cultivar Jinmai39 was used to investigate the transcription of *TaTPPs* in the presence of salt, drought, and ABA treatments. The seedlings were grown in a growth chamber at 22 °C with 16 h/8 h of light/ darkness and a light intensity of 9000 lux. Wheat plants were treated with either double-distilled water (control) or a 20% PEG-6000 or a 250 mM NaCl solution at the 2–3 leaf stage for drought and salt stress, respectively. For abscisic acid (ABA) treatment, plants at the same stage are sprayed with 100 mM abscisic acid (ABA) or 0.1% (*v*/*v*) ethanol (control). To analyze the expression of *TaTPPs* during leaf senescence, the delayed senescence wheat cultivar Yannong19 was grown in field conditions and collected samples from flag leaf at 0, 7, 10, 16, 19, 22, 24, and 25 days after anthesis. All the leaves after collection are immediately frozen into liquid nitrogen and stored at −80 °C for further RNA extraction.

### 2.7. RNA Extraction, Quantitative Real-Time Reverse Transcription PCR Analysis and Protein Interaction Network

The Quick RNA isolation Kit (Huayueyang Biotechnology, Beijing, China) was used to extract RNA according to the manufacturer’s instructions and DNase I treatment was used to remove DNA contamination. The RevertAid First Strand cDNA Synthesis Kit (Thermo Scientific, Waltham, MA, USA) was used to synthesize cDNA from a 3-µg aliquot of total RNA from each sample. To measure the expression of *TaTPPs* qRT-PCR analysis was performed with specific primers (Table S1), as described previously [65]. The ABI PRISM 7500 system (Applied Biosystems, Foster City, CA, USA) was used to generate threshold values (CT) and the transcription level of *TaTPPs* was measured using the comparative 2^−∆∆CT^ technique that was standardized with the *Elongation factor 1α* (*TaEF-1α*) (GenBank accession no. Q03033) [66,67] (Table S1). All of the studies were carried out three times. The TaTPP protein interaction network was examined using the STRING online server (https://string-db.org/, accessed on 27 April 2021).

## 3. Results

### 3.1. Identification and Annotation of Wheat TPPs

We identified a total of 31 TPP protein sequences in the wheat genome (Table 1 and Table S2). This number is relatively large when compared to TPPs previously identified in *Arabidopsis*, rice, and maize (Table S3). Wheat has a greater ploidy level and a larger genome size as it originated from the natural hybridization of three closely related genomes (A, B, and D), which may justify this result [68]. These protein sequences were encoded by 31 genes, three of which were chosen as representatives because they showed splice variants with full domains. A detailed description of *TaTPPs* is summarized in Table 1. The ORF of *TaTPPs* ranged from 750 to 1755 bp, with protein lengths ranging from 249 to 584 amino acids (Table 1). The molecular weight of the genes ranged from 28.67 KDa to 65.02 KDa. (Table 1). Fifteen genes were found to be basic (>7) and 16 genes were found to be acidic (<7) based on the predicted pI value (Table 1).

In addition, the Aliphatic Index and Instability Index were computed. The Aliphatic Index measures how much space is taken up by aliphatic side chains in Alanine, Isoleucine, Leucine, and Valine amino acids [69]. The Aliphatic Index ranges observed were 72.28 to 86.42, and the Instability Index ranges were 32.59 to 55.74 (Table 1). The high Aliphatic Index of a protein sequence suggests that it can function at a broad range of temperatures, whereas the Instability Index shows whether the protein is stable or unstable [70]. All the TaTPPs had negative GRAVY values ranging from −0.700 to −0.142 (Table 1). A protein with a negative GRAVY value is non-polar and hydrophilic in nature [69].

### 3.2. Subcellular Localization Prediction and Chromosomal Distribution of TaTPPs

**Figure 2:**
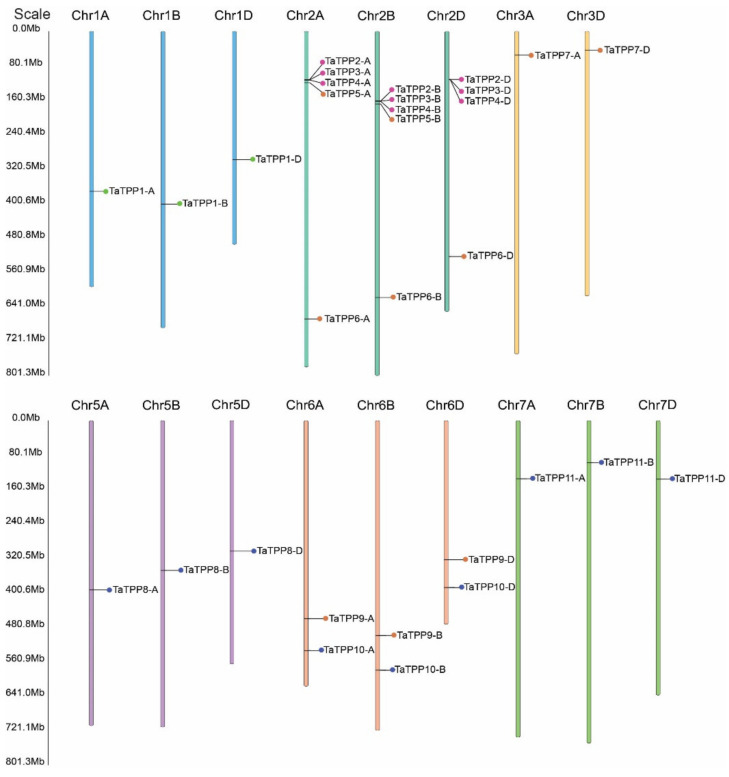
Graphical presentations of *TaTPPs* chromosomal distribution of on wheat chromosomes. The name of the gene on the right side and the location of the *TaTPPs* is indicated by the colored circular circle on the chromosomes. The three subgenomes chromosomal numbers are shown at the top of each bar.

PredSL (http://aias.biol.uoa.gr/PredSL/index.html accessed on 22 September 2021) was used to predict subcellular localization. Subcellular localization of the TaTPPs was predicted mostly in the chloroplast, whereas, TaTPP1-A, TaTPP7-D, TaTPP10-B appeared to be localized in the mitochondrion (Table 1). Moreover, TaTPP5-B, TaTPP7-A were predicted as secreted proteins (Table 1). However, TaTPP5-A, TaTPP10-A, TaTPP10-D were predicted with unknown localization (Table 1). A schematic diagram was created to explain the chromosomal location of *TaTPPs*. The *TaTPPs* are present on 17 wheat chromosomes (Figure 2 and Table 1). On the chromosomes of the A subgenome, the highest number of *TaTPP* genes (11 genes) were mapped. B and D subgenomes had 10 *TaTPP* genes in each subgenome. The maximum 14 genes of *TaTPPs* were located on chromosome 2 (Figure 2). Chromosome 6A, 6B and 6D, had 2 genes on each chromosome and 1A, 1B, 1D, 3A, 3D, 5A, 5B, and 5D had only a single gene. On the other hand, no *TaTPPs* were found on chromosomes 3B, 4A, 4B, or 4D (Figure 2 and Table 1), suggesting that *TPP* family genes were unevenly distributed throughout the three subgenomes of wheat.

**Figure 3:**
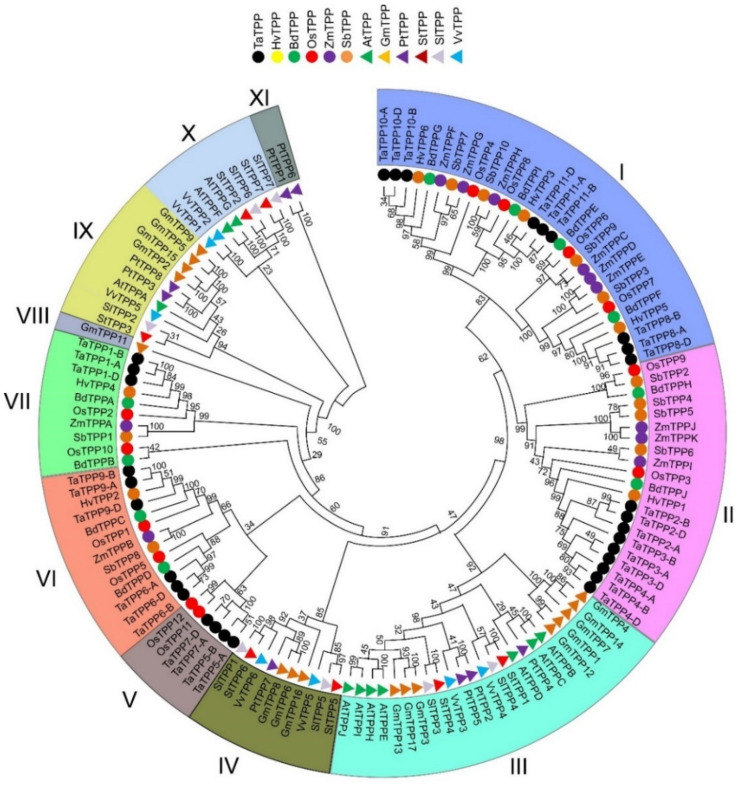
Phylogenetic analysis of TaTPP proteins. The tree was generated using MEGA X by the maximum likelihood method with 1000 bootstrap values. All the species and protein ID used for constructing tree were presented in Table S6.

We further investigated the duplication events in the *TaTPP* gene family in the context of wheat being hexapolyploid and having big genomes. Genes are usually considered duplicated when the query cover and identity value of gene sequences are more than 80% [71]. It has also been reported that genes are considered duplicated when protein sequence similarity and identity are more than 70% and 75%, respectively [72]. By analyzing the sequences, we found 27 pairs of *TaTPPs* with a sequence identity ranges from 82.14% to 95.25% and 100% query cover within all gene pairs (Tables S4 and S5) and identified in the same phylogenetic tree clade (Figure 3). We further computed the non-synonymous (Ka) and synonymous (Ks) substitutions, as well as the Ka/Ks ratios, for the 27 *TaTPP* gene pairs to determine the selection pressure on the duplicated *TaTPPs* (Table S5). These gene pairs Ka/Ks ratios were smaller than one, indicating that they developed under functional restriction with negative or purifying selection. The divergence period ranged from 2.93 to 13.3 million years ago (MYA), showing that these gene pairs were duplicated recently (Table S5).

### 3.3. Phylogenetic and Conserved Domain Analyses of TaTPP Proteins

A phylogenetic tree containing full length TPP protein sequences from twelve plant species was constructed by the maximum likelihood method to better understand the evolutionary relations among the TaTPP proteins with other species (Figure 3, Table S6), including five species from monocot: *Hordeum vulgare*, *Brachypodium distachyon*, *Oryza sativa*, *Zea maize*, *Sorghum bicolor*; and 6 species from dicot: *Arabidopsis thaliana*, *Glycine max*, *Populus trichocarpa*, *Solanum tuberosum*, *Solanum lycopersicum*, *Vitis vinifera*. The results indicated that TPP proteins were divided into eleven clades, where clade I was the largest with 30 members. Clades II to XI (total 10 clades in order) included 21, 24, 10, 6, 13, 10, 1, 10, 8, and 2 members, respectively (Figure 3).

Plants classified as dicots and monocots were divided into distinct clades. Proteins from monocot plants were grouped into clade I, clade II, clade V, clade VI, and clade VII, whereas proteins from dicot plants were grouped into clade III, clade IV, clade VIII, clade IX, clade X, and clade XI. The highest number of TaTPP proteins were grouped into clade I and clade II, which had nine proteins in each clade. In addition, clades V, VI, VII contained four, six, and three TaTPP proteins, respectively (Figure 3). Most of the wheat TPP proteins were closely related to *H. vulgare*, *B. distachyon*, and *O. sativa*, suggesting their conserved function with those plant species and offering information that can be used to conduct a more in-depth functional analysis. All the *TaTPPs* were assembled into 11 groups, as sequences from A, B, and D subgenome of 11 groups clustered together in the phylogenetic tree (Figure 3) and protein sequence identity was more the 88% between A, B, and D subgenome of each group (Table S4). Thus, we considered the protein sequences from A, B, and D subgenome of each group are copies of separate *TaTPP* genes and named them according to the ascending order of the chromosomal location (Table 1).

**Figure 4:**
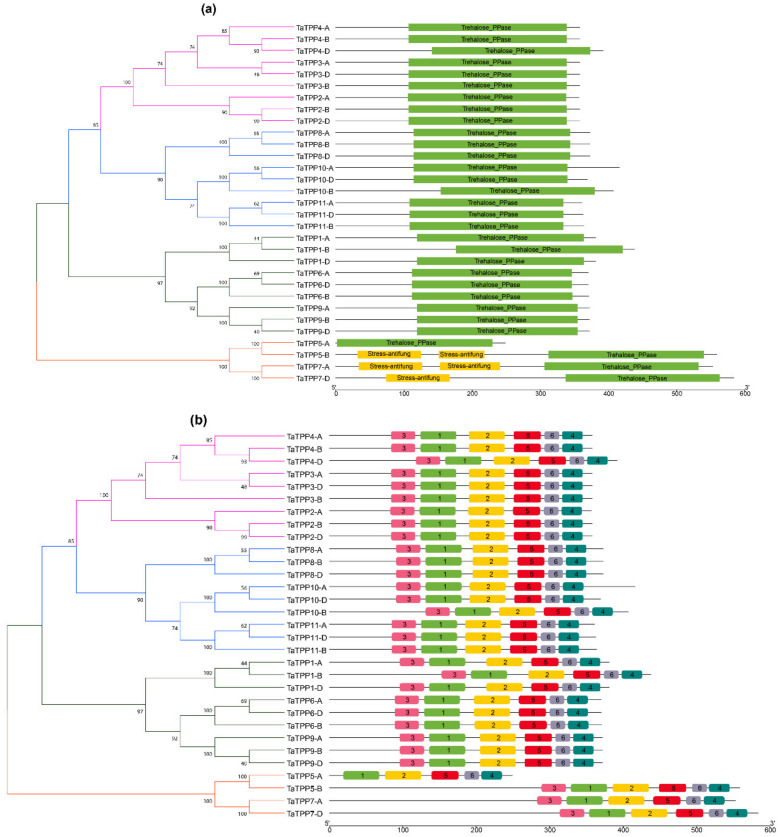
Conserved domain and motif of TaTPPs. (**a**) The conserved domain of TaTPP members was identified from Pfam and SMART databases and presented using TBtools. (**b**) The conserved motifs of TaTPP members. Six motifs were identified using MEME program and presented with different colored boxes.

Further, the Pfam database was utilized to find the important component domains of TaTPP proteins [59]. All the TaTPP proteins contain a specific Trehalose PPase domain (PF02358). In addition, a stress antifungal domain was found in TaTPP-5A, TaTPP7-A and TaTPP7-D (Figure 4a). We used MEME suite 5.1.1 to evaluate motif sequences for 31 TaTPPs and found six significant motifs (motifs 1–6) (Figure 4b). All the motifs were found to be conserved in all TaTPP proteins except for TaTPP5-A, which lacks lacks motif 3 (Figure 4b).

### 3.4. Gene Structure and Evolution Analyses of TaTPPs

**Figure 5:**
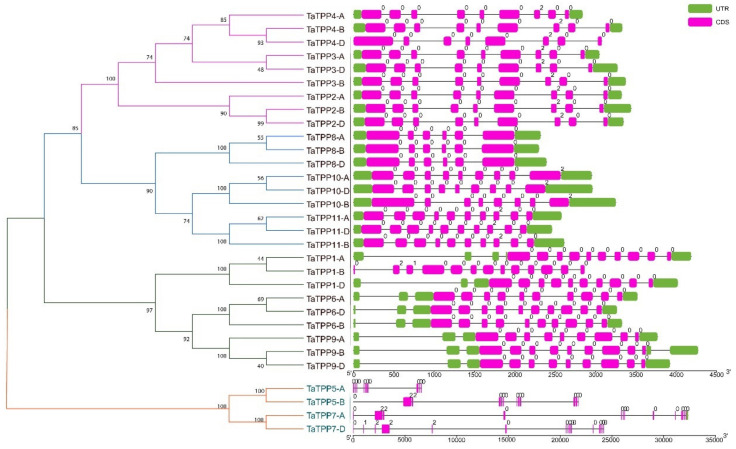
Structural organizations of *TaTPPs*. The introns are shown by black lines, whereas the exons are represented by pink boxes and untranslated regions (UTRs) are represented with green boxes. Intron phase, 0: phase 0, 1: phase 1 and 2: phase 2 denotes that a codon is not disrupted by introns, a codon between the first and second bases is disrupted by an intron and a codon between the second and third bases is disrupted by an intron, respectively.

The exon-intron structures of *TaTPPs* were studied to better understand their structural features (Figure 5). The *TaTPP* gene family had a lot of variation in terms of gene structure, according to gene structure analyses as introns ranged from 4 to 13. Most of the *TaTPPs* contain eight or nine introns. A maximum of 13 introns was found in *TaTPP7-D* and a minimum of four introns were observed in *TaTPP8-B* (Figure 5). Moreover, different *TaTPPs* showed different intron phase patterns. *TaTPP1-A*, *TaTPP1-D*, *TaTPP5-A*, *TaTPP6*, *TaTPP8*, *TaTPP9* showed phase 0 and *TaTPP2*, *TaTPP3*, *TaTPP4, TaTPP5-B*, *TaTPP7-A*, *TaTPP10*, *11* showed phase 2 patterns, whereas *TaTPP1-B* and *TaTPP7-D* exhibited all phases (Phase 0,1,2) (Figure 5).

**Figure 6:**
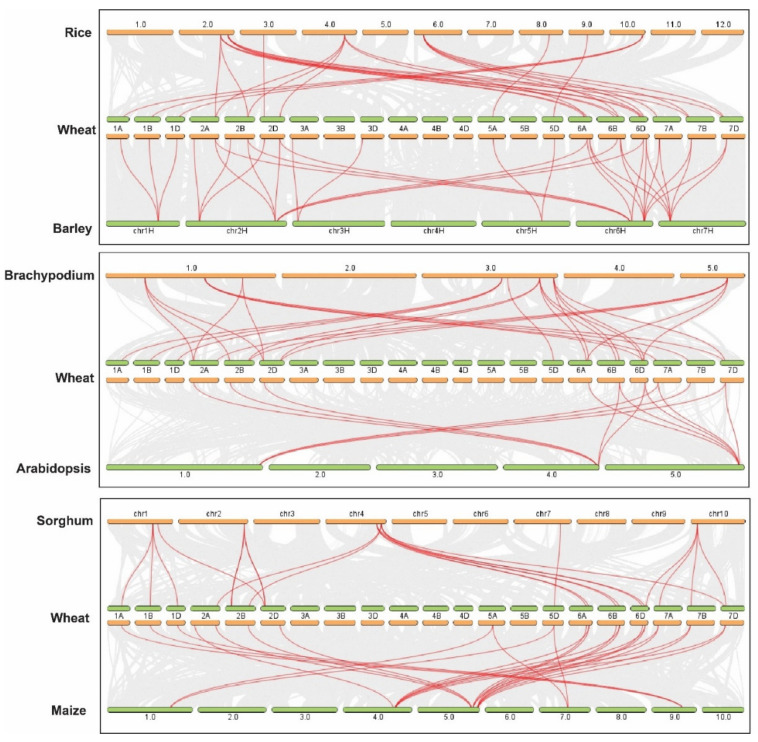
Syntenic relationship between *TaTPPs* with rice, *Arabidopsis*, *Brachypodium,* sorghum, and maize. The collinear blocks within wheat and other plant genomes are shown by gray lines in the background, while the syntenic *TaTPP* gene pairs are highlighted by red lines.

Further, the Multiple Collinearity Scan toolkit was used to investigate the synteny networks between *TaTPPs* and other wheat relatives and model plants. The results showed that 27, 26, 13, 33, 26, and 22 orthologous gene pairs were identified between *TaTPPs* and other *TPPs* in *B. distachyon*, *O. sativa*, *A. thaliana*, *H. vulgare, Z. mays*, and *S. bicolor*, respectively (Figure 6 and Table S7). A collinear relation was observed for 19, 18, 9, 22, 17 and 19 *TaTPPs* with other *TPPs* in *B. distachyon*, *O. sativa*, *A. thaliana*, *H. vulgare*, *Z. mays*, and *S. bicolor*, respectively. *TaTPP6*, *TaTPP9*, *TaTPP10* and *TaTPP11* were shown to have more than one pair of orthologs. Thus, these *TaTPPs* might have a crucial role in the evolution of *TPPs*. These findings imply that *TaTPPs* in wheat may have evolved from other plant species orthologous genes.

### 3.5. 3-D Protein Structure Analysis

The 3-D structure reveals a few key residues linked to biological processes or intended outcomes [73]. Thus, we used SWISS-MODEL to identify the 3-D model of TaTPP proteins (Figure S1a). For all TaTPP proteins, the 3-D structures were analyzed using template “5gvx.1.A.” and predicted 3-D structures covering the N-terminus and C-terminus regions of 31 TaTPP proteins (Figure S1a). Within 4 A°, three conserved residues that worked as ligands were identified. The interaction of those ligands with chain A and the magnesium ion (Mg^2+^) indicates that TaTPP proteins have distinct catalytic activities, which have also been reported for AtTPP, ZmTPP, ScTPP, CaTPP, and EcTPP that have a catalytic function and they are all similar to one other by 80% [39,74]. Further, we used SOPMA to calculate the secondary structure elements of protein sequences (Table S8). TaTPP proteins were found to contain a range of 35.70% to 47.99% α helix, 13.41% to 18.18% extended strand, 6.62% to 9.93% β turn and 8.38% to 41.21% random coil (Table S8). All TaTPPs except TaTPP5, TaTPP7, TaTPP9-A, and TaTPP10-B had a coiled coil-like structure in the C-terminus and one Mg^2+^ ligand each was observed in all the TaTPPs (Figure S1a).

To validate TaTPP protein structures, we employed SWISS-MODEL analysis and the MolProbity server (Figure S1b and Table S9). The produced Ramachandran plot has an average favored region of 94.07%, an average allowed region of 99.08%, and an average outer region of 0.91% (Table S9). The average sequence identity was 34.95%, with a similarity of 37%, covering 68% of the query sequences obtained by the X-ray Method in 2.6 A° (Table S9). The ligand interaction between chain A and Mg^2+^ was confirmed with the Protein–Ligand Interaction Pipeline (PLIP), and the residue site was noticed to be highly conserved. We investigated these conserved residues further in all TaTPP protein sequence alignments and found that they include aspartic acid (D/Asp), which is conserved in motif 3 and motif 6. (Figure S2, Table S9). For improved visual clarity, the side chains of the catalytic triads were expanded with the TaTPP1-A residues (Figure S3).

### 3.6. Analysis of Cis-Regulatory Elements

**Figure 7:**
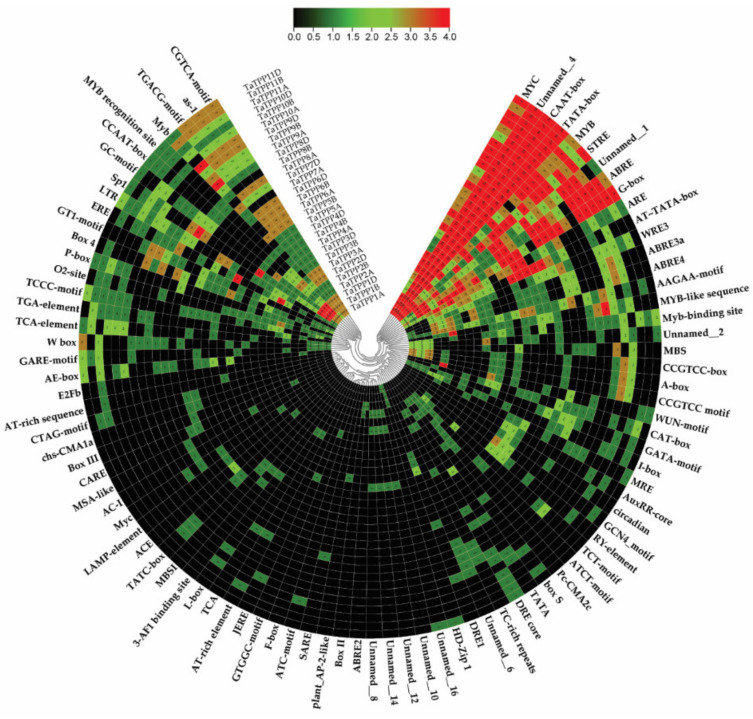
Putative *Cis*-acting regulatory elements (CREs) of *TaTPPs*. The CREs were identified with the 2 kb upstream sequences of the start codon using the PlantCARE online server and presented using TBtools. Red color indicates the CREs with high frequency, while black color indicates CREs with zero frequency.

To examine the responses of *TaTPPs* members to various stimuli, the 2 kb promoter sequences upstream of the start codon of these genes were submitted to the PlantCARE service to predict their *Cis*-regulatory elements (CREs). A total of 90 CREs with a frequency of 1985 were identified in all *TaTPP* promoters (Figure 7, Table S10). Among them, 72 CREs were related to phytohormones, stress, growth, and development (Figure 7, Table S10). All of the identified CREs were divided into five groups according to their known functions (Table S10 and Table S11). Group I contained four core *Cis*-elements, including AT~TATA-box, CAAT-box, TATA, TATA-box. TATA-box (which comprises TATA and AT TATA-box) is a critical promoter element found in approximately 30% of transcription start sites and the CAAT-box is a kind of promoter that may influence the choice of transcription start location [75]. TATA-box and CAAT-box are generally present 25–30 bp and ~75 bp upstream of the transcription start site, respectively, and both of them are found in a wide range across all the promoters.

Group II contained 44 stress-related CREs, among them 20 were light-responsive *Cis*-elements such as 3-AF1 binding site, 3-AF1 binding site, ABRE4, ACE, ATCT-motif, Box 4, AE-box, Box II, LAMP-element. The stress-responsive CREs consist of one anaerobic-responsive element (ARE), one low-temperature-responsive element (LRT), one drought-responsive element (MBS), two wound-responsive elements (WUN-motif, box S), one cold- and dehydration-responsive (DRE core) and 17 defense- and stress-responsive elements (as-1, TC-rich repeats, W box, CCAAT-box, MYB, MYB recognition site, Myb, Myb-binding site, MYB-like sequence, MYC, Myc, STRE, WRE3, Unnamed_1, Unnamed_8, GC-motif, AT-rich sequence). There were 12 CREs in group III, involved in cell development including seed specific expression (AAGAA-motif, RY-element), cellular development and cell cycle regulation (AC-I, MSA-like), meristem expression (CAT-box, CCGTCC-box, CCGTCC-box), circadian control (circadian), differentiation of palisade mesophyll cells (HD-Zip I), cell cycle regulation (MSA-like), and endosperm expression (GCN4_motif).

Additionally, the hormone-responsive CREs in group IV included 16 CREs such as abscisic-acid-responsive element (ABRE, ABRE2), auxin-responsive elements (AuxRR-core and TGA-element), salicylic-acid-responsive element (TCA-element, TCA, SARE), methyl-jasmonate-responsive elements (TGACG and CGTCA motifs), ethylene-responsive element (ERE) and gibberellin-responsive elements (GARE-motif, P-box, and TATC-box). There were also 14 CREs in group V with unknown functions. CTAG-motif and A-box might act as a CRE, Unnamed_2 might act as an antisense transcript, BOX III might function as a protein binding site and Unnamed_16 was found to be involved in sugar transporter family genes. Most *TaTPPs* possessed one or more CREs associated with hormone and stress-related activities, suggesting that *TaTPPs* may be engaged in a variety of physiological processes as a result of diverse environmental adaptations.

### 3.7. Transcriptional Patterns of TaTPPs in Different Organs and Developmental Stages of Wheat

**Figure 8:**
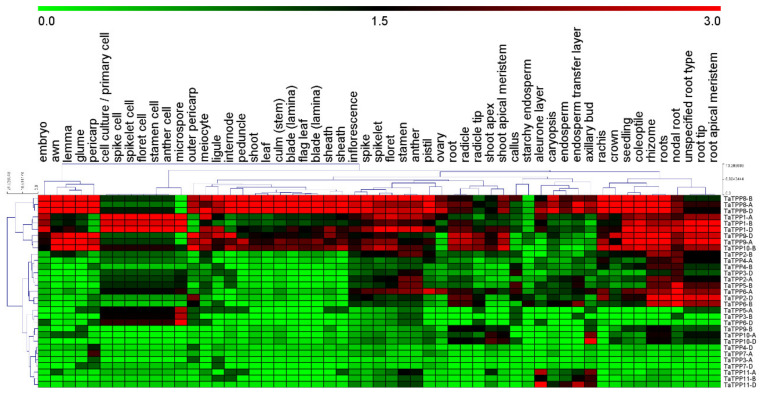
Transcription profiles of *TaTPPs* in different wheat organs. mRNA transcription data of *TaTPPs* in different wheat organs were retrieved from Genevestigator and presented using MeV software.

To investigate the transcription level of *TaTPP* genes in different wheat organs and development stages, mRNA transcripts data was collected from Genevestigator and visualized with a heatmap in Figure 8 and Figure S4. The transcript data were divided into six groups. Group I included callus, Group II included primary cells (cell culture, spike cell, spikelet cell, floret cell, stamen cell, anther cell, meiocyte, microspore), Group III included seedlings (seedling, coleoptile, root, radicle, radicle tip), Group IV included inflorescence (inflorescence, spike, rachis, spikelet, floret, stamen, anther, pistil, ovary, lemma, awn, glume, caryopsis, embryo, endosperm, aleurone layer, starchy endosperm, endosperm transfer layer, pericarp, outer pericarp), Group V included shoot (shoot, culm (stem), internode, peduncle, leaf, blade (lamina), sheath, flag leaf, blade (lamina), ligule, sheath, crown, shoot apex, shoot apical meristem, axillary bud) and Group VI included rhizome (rhizome, roots, nodal root, unspecified root type, root tip, root, apical meristem). Our results showed that *TaTPP1*, *TaTPP8*, *TaTPP9-A*, *TaTPP9-D* and *TaTPP10-B* had the highest transcriptions in most of the organs compared to other *TaTPPs* (Figure 8). In addition, high expression was observed for *TaTPP2-D*, *TaTPP5-B*, *TaTPP6-A* and *TaTPP6-B* only in the rhizome group. In contrast, other *TaTPPs* had no expression in most of the organs (Figure 8).

Further, we observed the mRNA transcripts level of *TaTPPs* during different developmental stages of wheat, such as germination, seedling growth, tillering, stem elongation, booting, inflorescence emergence, anthesis, milk development, dough development, and ripening (Figure S4). A number of *TaTPPs* were expressed differently at various stages of wheat development. For example, *TaTPP8* and *TaTPP4-A* were found to be expressed in all stages, whereas *TaTPP1* and *TaTPP9* were induced in all except the ripening stage. *TaTPP4-D* was expressed in all except stem elongation and *TaTPP3-D* was expressed in all except tillering and ripening stages. *TaTPP5* and *TaTPP7* showed very low expression in all wheat developmental stages and other *TaTPPs* were either slightly or highly expressed in one or more developmental stages (Figure S4). These findings suggest that various *TaTPPs* may have a role in the development of various tissues at different development stages.

### 3.8. Transcriptions of TaTPPs Were Induced in Response to ABA, Abiotic Stresses and Leaf Senescence

**Figure 9:**
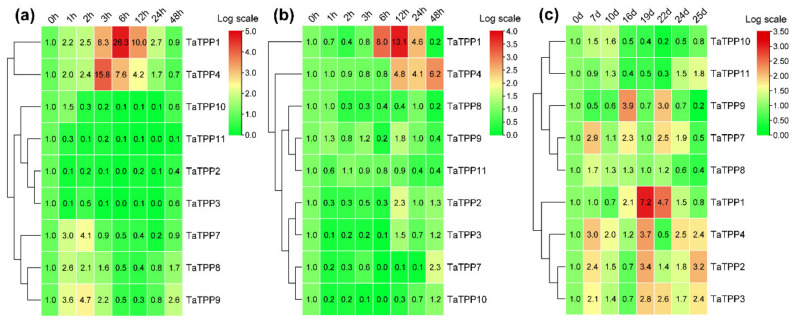
Relative transcript profiles of *TaTPPs* in response to (**a**) abscisic acid (ABA), (**b**) drought stress and (**c**) leaf senescence. The relative transcripts of all genes were analyzed using qRT-PCR. The relative transcript levels of *TaTPPs* were measured using the comparative threshold (2^−ΔΔCT^) method. Data normalized with the transcripts of wheat elongation factor, *TaEF-1α*. The 0 h post treatment (**a,b**) or 0 days after anthesis (**c**) was used as a control and standardized with 1. Red and green colors denote strong and weak transcription of *TaTPPs*, respectively. The heat map was generated with TBtools and tree was constructed with the average linkage clustering method.

Wheat seedlings treated with ABA or abiotic stress (drought and salinity) were used to analyze the transcriptional pattern of the *TaTPPs* in wheat. Under ABA treatment, *TaTPP1* and *TaTPP4* exhibited upregulated transcriptions at most of the time points and significant upregulation was observed from 3 to 12 hpt (hours post treatment). Moreover, three *TaTPPs* (*TaTPP7*, *TaTPP8* and *TaTPP9*) were upregulated immediately after ABA treatment and transcriptions decreased with an increase in ABA treatment time points. Transcriptions were significantly downregulated for *TaTPP2*, *TaTPP3* and *TaTPP11* at most of the time points after ABA treatments compared to control (0 hpt) (Figure 9a). The transcriptional patterns of *TaTPP* members were examined following drought stress in wheat to provide insight into the underlying functional roles of wheat *TPPs* in response to drought stress. During the drought stress treatment, only *TaTPP1* and *TaTPP4* showed significant upregulations at a later time post treatment. A slight upregulation or significant downregulation was observed for all other *TaTPPs* after drought stress in wheat (Figure 9b). The transcriptional levels of *TaTPP* members were examined to elucidate the mechanism of gene responses to leaf senescence in wheat. Most of the *TaTPP* members were slightly or highly induced during leaf senescence, *TaTPP1* showed obvious upregulated transcriptions at 19 and 22 days after anthesis compared to the control (0 days after anthesis) (Figure 9c).

**Figure 10:**
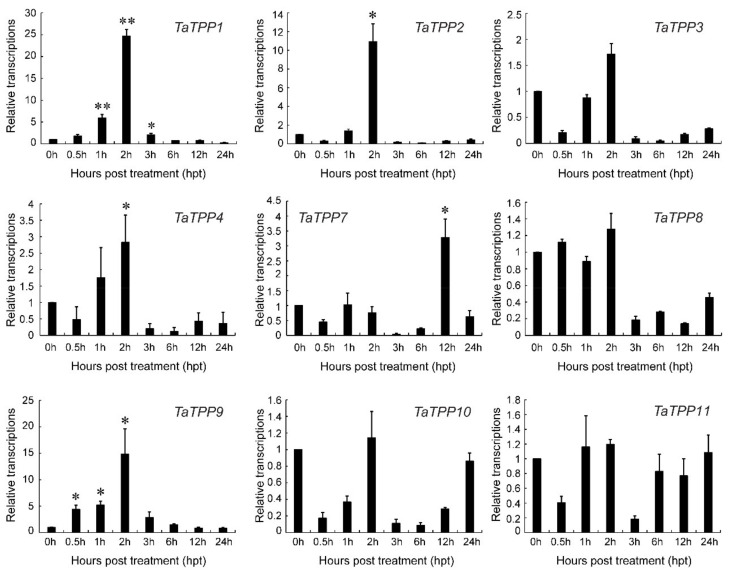
Relative transcript profiles of *TaTPPs* in response to salt stress. The relative transcripts of all genes were analyzed using qRT-PCR. The relative transcript levels of *TaTPPs* were measured using the comparative threshold (2^−ΔΔCT^) method. Data normalized with the transcripts of wheat elongation factor, *TaEF-1α*. The 0 h post treatment was used as a control and standardized with 1. Values represent the mean ± SD from three independent biological samples. Asterisks (*p* < 0.05) or double asterisks (*p* < 0.01) designate significant differences from 0 hpt by the Student’s *t*-test.

Further, we analyzed the transcriptions of *TaTPP* members under salt stress by qRT-PCR to observe the involvement of *TaTPPs* in wheat salt tolerance (Figure 10). Significant upregulation of the transcripts was observed for *TaTPP1*, *TaTPP2*, *TaTPP4* and *TaTPP9* at an early stage of salt treatment and downregulations were observed at the later stage of salt treatment. Moreover, *TaTPP7* showed a significant upregulation only at 12 h post treatment (hpt) compared to the control (0 hpt). In contrast, either no changes or significant downregulations were observed for other *TaTPP* members compared to the control (Figure 10). However, no expression was observed for *TaTPP5* and *TaTPP6* by qRT-PCR in all aspects. Overall, these findings suggest that *TaTPPs* act as an important regulator of wheat abiotic stress and leaf senescence responses.

### 3.9. Protein–Protein Interaction Analysis of TaTPPs

The STRING database was used to build a network to study protein–protein interactions between TaTPPs and other wheat proteins (Figure S5 and Table S12). From prediction results, it was found that TaTPPs can interact with five other wheat proteins. Traes_1AL_7531AC097.1, Traes_1BL_2AE952A77.1 and Traes_1DL_50B29C62B.2 have encoded an enzyme called TRE, which is hydrolyzed Trehalose to synthesize two molecules of glucose. Moreover, Traes_6DL_33F8A5EF4.1 has encoded TPS enzyme which produces T6P, a phosphorylated intermediate, from UDPG and G6P and Traes_4AS_4B8E78B13.1 was an unknown protein. Thus, our results suggesting that TaTPPs might interact with other enzymes that are involved trehalose biosynthesis pathway to accelerate the trehalose biosynthesis process.

## 4. Discussion

The *TPP* gene family has been characterized as catalytic enzymes that mainly function in trehalose biosynthesis [18,76,77]. Despite their catalytic function, a portion of *TPP* genes has been identified to be involved in growth and development, response in abiotic and biotic stress and senescence [27,28,29,31,32,33,35,42,78,79]. Although wheat is one of the most economically important cereal crops, systemic studies on *TPP* homologs in wheat have not been reported yet.

In the present study, we analyzed wheat *TPPs* with other species and identified 31 *TPPs* in wheat based on the Chinese Spring genome sequence (Table 1). The highest number of *TPPs* were found in wheat and these genes were distributed over 17 chromosomes (Figure 2). In comparison to previously described *TPPs* in *Arabidopsis*, rice, maize, and purple false brome, the wheat *TPP* gene family has been significantly extended with relatively more *TPPs* [38,74,80,81,82]. The major driving forces for extending the gene family in various plant species are gene duplication mechanisms, which include segmental, tandem, and whole-genome duplications [83,84]. All the *TaTPPs* are distributed unevenly on the wheat chromosome and the number ranges from 1 to 5 on each chromosome (Figure 2). Gene duplication analysis revealed that 27 pairs of *TaTPPs* duplicated within the wheat genome (Tables S4 and S5). The gap between genes on the chromosomal map of common wheat was higher than 200 kb (Figure 2), indicating that these genes were not formed via tandem duplication [85]. In addition, Ka/Ka ratio was less than one for all pairs of duplicated genes, suggesting that *TaTPPs* were subjected to a rigorous purifying selection (Table S5) and a comparable segmental duplication event was also observed for *TPPs* in rice [74]. Natural whole-genome duplicating processes might have led to the expansion of the *TaTPP* gene family. Thus, these findings suggest that whole-genome and segmental duplications might be vital in the expansion and evolution of *TaTPPs*.

Phylogenetic analysis of 31 TaTPP proteins and 11 other plant species showed that these proteins clustered into 11 groups, where TPPs from monocots and dicots species were grouped into separate clades (Figure 3). TaTPP proteins were grouped into clade I, clade II, clade V, clade VI, and clade VII and closely related to *Brachypodium*, rice, and barley TPPs, suggesting that TaTPP proteins might originate from a common ancestor. TaTPP5 and TaTPP7 have moved far away from the cluster of all other TPPs in the radiation tree (Figure S6) that was similar to OsTPP11 and OsTPP12 as previously reported [74]. The *TaTPP* gene structure study demonstrated that the majority of *TaTPPs* had highly conserved gene structures. The size of an intron has a significant impact on the size of a gene. The number of introns in *TaTPPs* ranged from 4 to 13 and most of the *TaTPPs* had 8 or 9 introns (Table 1). The difference in total intron length between the largest gene *TaTPP7-A* (32 kb) and the shortest gene *TaTPP-8B* (2.3 kb), resulted in a significant variation in gene size. Further, multiple alignments of TaTPP protein sequences revealed that the Trehalose_PPase domain and conserved motif are conserved within the *TaTPPs* (Figure S2). Among the identified six motifs, all the motifs were highly conserved in all *TaTPPs* except *TaTPP5-A*, which lacks motif 3. All the *TaTPPs* had a complete Trehalose_PPase domain, suggesting the various proteins’ functional equivalence and evolutionary relationships. In addition, *TaTPP5-B* and *TaTPP7* had a stress-antifungal domain which has been reported to be involved in disulphide bridges and response to salt stress [86,87]. Subcellular localization prediction showed that most of the TaTPPs are localized in the chloroplast, whereas some of them are found in the mitochondrion or secreted protein (Table 1). In *Arabidopsis* or rice, different localizations were also detected. For instance, AtTPPD and AtTPPE were localized in the chloroplast whereas AtTPPA, AtTPPB, AtTPPC, AtTPPF, and AtTPPH were found in the cytosol and AtTPPG, AtTPPI, and AtTPPJ showed localization in the nucleus [88]. This variation in the localization of TaTPPs might be due to a lack of conserved N-terminus (Figure S2). According to the 3-D structure analysis, all TaTPPs were highly conserved and showed Mg^2+^ ligand-binding sites in SWISSMODEL (Figure S1a), which are shown to have a role in catalysis by activating or inhibiting a variety of enzymes [89,90]. To investigate the *TPP* gene synteny relationship in wheat and other plant species, we identified 27, 26, 13, 33, 26 and 22 orthologous gene pairs between *TaTPPs* and other *TPPs* in *B. distachyon*, *O. sativa*, *A. thaliana*, *H. vulgare*, *Z. mays*, and *S. bicolor*, respectively (Figure 6 and Table S7). These findings imply that *TaTPPs* in wheat might have evolved from other plant species orthologous genes.

A non-coding DNA sequence found in the promoter region of a gene is known as a CREs. Different CREs distribution in promoter regions may indicate variations in gene regulation and function [91]. To identify the CREs, we used 2kb promoter regions of all *TaTPPs* and classified into five groups according to their known functions (Figure 7, Table S10). Stress related CREs were identified in high frequency compared to cellular development and hormone related CREs, suggesting the involvement of *TaTPPs* in response to stress. ABRE, At~ABRE, ABRE3a, and ABRE4 are ABA-responsive CREs that play important roles in seed dormancy, stomatal closure, leaf senescence, and plant biotic and abiotic stress responses. Multiple ABREs or their combinations have been reported to act as CEs (Coupling Elements) in the formation of ABA-responsive complex (ABRC) [92,93,94,95,96,97]. The ABRE CREs were predicted in all *TaTPPs* promotor with high frequency and ABRE3a and ABRE4 CREs were found in most of the *TaTPPs* except *TaTPP5*, *TaTPP7*, *TaTPP9* and *TaTPP11*. Following that, we also discovered TGA-element, and AuxRR-core (auxin-responsive element), TCA-element (salicylic acid responsiveness), CGTCA-motif (MeJA responsiveness) and *p*-box and TATC-box (gibberellin responsive element), among other hormone-related CREs [98], that might potentially induce possible signal transduction pathways for wheat *TPPs* during stress response. Furthermore, other CREs linked to a variety of development and stress were also predicted in *TaTPP* promoters with high frequency, including MBS (drought inducibility), MYC (drought-responsive CRE) MYB and STRE (stress response element), as-1(Defense response), Unnamed_1 (ABRE-like CRE, responsible for biotic and abiotic stress responses), ARE (anaerobic induction CRE), Unnamed_4 (might responsible for tissue specific expression) and AAGAA-motif (involved in seed specific expression) [91]. These findings suggest that *TPP* gene family members in wheat may be controlled by a variety of developmental events, hormones, and stress; however, additional experimental investigations will be required to validate this.

Higher transcriptional levels of *TaTPPs* were observed in different wheat organs and developmental stages. *TaTPP1*, *TaTPP8* and *TaTPP9* were expressed in most organs and developmental stages but predominantly expressed in the roots, suggesting that they could be important for root physiology. (Figure 8, Figure S4). Previous evidence showed that *AtTPPE* modulates ABA-mediated root growth and a rice *TPP*, *OsTPP7*, enhanced the anaerobic germination [41,99]. Wang et. al. [27] reported that seed germination was regulated by *OsTPP1* via crosstalk with the ABA catabolism pathway. In addition, high expression was also observed for *TaTPP1*, *TaTPP8* and *TaTPP9* in all developmental stages except dough development and ripening, suggesting that these genes might have a significant association with wheat developmental processes. Plants have developed sensory and response systems that enable them to adjust physiologically to environmental stress conditions such as drought, excessive salt, and low temperature stress. Previous studies in rice and *Arabidopsis* demonstrated the involvement of *TPP* genes in various environmental stresses and ABA signaling [32,37,38,40,41,100]. Under ABA treatment, *TaTPP1* and *TaTPP4* exhibited upregulated transcriptions at most of the time points and significant upregulations were observed from 3 to 12 hpt. Moreover, three *TaTPPs* (*TaTPP7*, *TaTPP8* and *TaTPP9*) were upregulated immediately after ABA treatment and transcriptions were decreased with the duration after ABA treatment and a significant downregulated transcription level was observed for *TaTPP2*, *TaTPP3* and *TaTPP11* at most of the time points after ABA treatment (Figure 9a). During the drought stress treatment, only *TaTPP1* and *TaTPP4* showed significant upregulations at later time post treatment. A slight upregulation or significant downregulation was observed for all other *TaTPPs* after drought stress in wheat (Figure 9b). An obvious significant upregulation was observed for *TaTPP1*, *TaTPP2*, *TaTPP4* and *TaTPP9* at an early stage of salt stress and downregulated transcriptions were observed at the latter stage of salt treatment. A similar expression pattern was also observed for rice *TPP* and *BdTPPC* genes that were upregulated in the first hour under abiotic stress [28,38]. Moreover, *TaTPP7* showed a significant upregulation only at 12 dpt. In contrast, either no changes or significant downregulation was observed for other *TaTPP* members compared to the control (Figure 10). The phenotype of mature *otsB*-overexpressing *Arabidopsis* plants included delayed senescence and decreased anthocyanin accumulation, suggesting that the role of *TPP* may perform a crucial role during leaf senescence in plants [42,43,44,45]. Our results showed that most of the *TaTPP* members were slightly or highly induced during leaf senescence, especially *TaTPP1* showed an obvious upregulated transcription (Figure 9c). Overall, these findings suggest that *TaTPPs* might act as an important regulator of wheat abiotic stress and leaf senescence responses and could be good candidate genes for wheat improvement under environmental stimuli. Moreover, protein network prediction revealed that TaTPP proteins possible interact with TaTPS or TaTRE protein which involved in trehalose biosynthesis pathway to accelerate the trehalose biosynthesis process (Figure S5).

**Figure 11:**
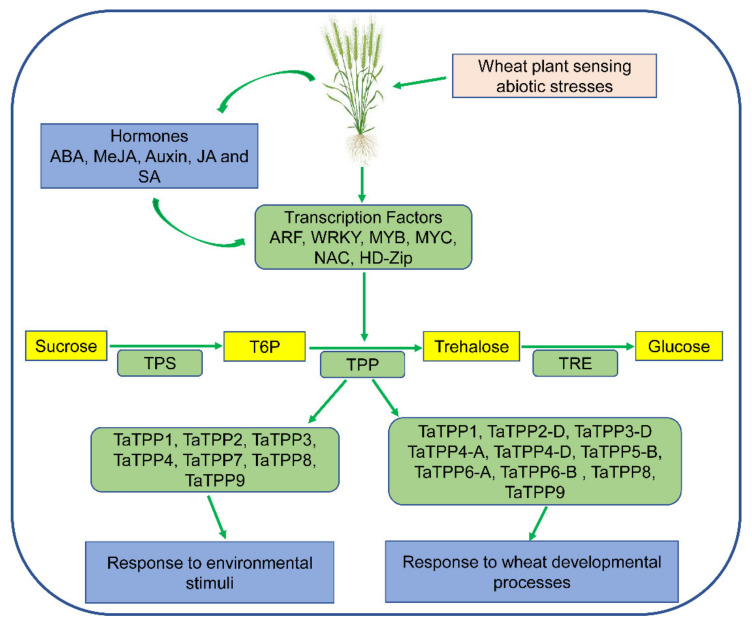
A proposed model for *TaTPP* genes functions in various wheat developmental processes and diverse stress conditions. ABA: Abscisic acid; MeJA: Methyl jasmonate; JA: Jasmonic acid; SA: Salicylic acid; ARF: Auxin response factors; MYB: Myeloblastosis; NAC: No apical meristem; TPS: trehalose-6-phosphate synthase; T6P: trehalose-6-phosphate; TPP: trehalose-6-phosphate phosphatase; TRE: trehalase. Yellow boxes indicate carbohydrates and light green boxes indicate proteins.

Furthermore, we suggested a feasible working model based on *TaTPPs* transcription profiling to illustrate the roles of *TaTPPs* in a range of biological processes in wheat (Figure 11). TPS produces T6P, a phosphorylated intermediate, from UDPG and G6P and then TPP dephosphorylates T6P to produce trehalose in the second phase. Trehalose is then hydrolyzed by an enzyme called TRE to synthesize two molecules of glucose [16]. The expression of *TaTPPs* was induced by both endogenous and exogenous stimuli in this model. These signals were detected by multiple *Cis*-regulatory elements, which then regulated the transcription and functions of *TaTPPs* involved in numerous plant developmental stages and stress situations, affecting plant growth and tolerance mechanisms (Figure 11).

## 5. Conclusions

In conclusion, a relatively comprehensive analysis of the *TaTPP* gene family was performed in this study, which may help to explain the biological activities of TaTPP proteins in developmental processes, stress responses and leaf senescence of wheat. However, our knowledge of their precise biological role is still lacking. Thus, in order to give important insights to help wheat breeders for developing resistant crops cultivars to unfavorable stress conditions, an extensive functional validation study of *TaTPPs* is necessary.

## Figures and Tables

**Table 1:** Detailed annotations of the *TaTPPs* in wheat.

Gene Name	Gene ID	Splice Variant	PC	ORF	Chromosome Location					Introns	Exons	Length (aa)	M.W. (KDa)	PI	Instability Index	Aliphatic Index	GRAVY	SL Prediction
					Chr	Strand	Start	End	Chr Length									
*TaTPP1-A*	TraesCS1A02G210400.1	1	VII	1146	1A	reverse	372639121	372643307	594102056	9	10	381	42.56	5.61	46.35	83.91	−0.314	chloroplast
*TaTPP1-B*	TraesCS1B02G224300.2	2	VII	1317	1B	reverse	402147526	402150391	689851870	12	13	438	49.46	6.09	47.92	86.32	−0.238	mitochondrion
*TaTPP1-D*	TraesCS1D02G213700.1	1	VII	1146	1D	reverse	298692275	298696295	495453186	9	10	380	42.54	5.53	45.29	84.13	−0.315	chloroplast
*TaTPP2-A*	TraesCS2A02G161000.1	1	II	1074	2A	forward	111921839	111925164	780798557	8	9	357	39.43	6.11	34.73	79.3	−0.254	chloroplast
*TaTPP2-B*	TraesCS2B02G187000.1	1	II	1077	2B	forward	161722263	161725704	801256715	8	9	358	39.62	6.04	35.64	82.6	−0.216	chloroplast
*TaTPP2-D*	TraesCS2D02G168100.1	1	II	1077	2D	forward	111588533	111591881	651852609	8	9	358	39.66	5.77	35.52	80.42	−0.249	chloroplast
*TaTPP3-A*	TraesCS2A02G161100.1	1	II	1077	2A	forward	112744717	112747766	780798557	8	9	358	39.63	7.14	35.67	79.61	−0.282	chloroplast
*TaTPP3-B*	TraesCS2B02G187100.1	1	II	1077	2B	forward	162007594	162010969	801256715	8	9	358	39.61	6.57	33.2	79.61	−0.264	chloroplast
*TaTPP3-D*	TraesCS2D02G168200.1	1	II	1077	2D	forward	112099169	112102442	651852609	8	9	358	39.50	6.84	34.77	79.08	−0.283	chloroplast
*TaTPP4-A*	TraesCS2A02G161200.1	1	II	1077	2A	reverse	113309228	113312068	780798557	8	9	358	39.63	6.77	35.05	79.86	−0.281	chloroplast
*TaTPP4-B*	TraesCS2B02G187200.1	1	II	1077	2B	reverse	162445597	162448929	801256715	8	9	358	39.65	7.12	32.59	79.86	−0.257	chloroplast
*TaTPP4-D*	TraesCS2D02G168300.1	2	II	1179	2D	reverse	112177996	112181078	651852609	7	8	392	43.58	6.44	39.59	82.65	−0.190	chloroplast
*TaTPP5-A*	TraesCS2A02G167100.1	1	V	750	2A	reverse	119307539	119314162	780798557	8	9	249	28.67	8.28	43.34	75.9	−0.700	other
*TaTPP5-B*	TraesCS2B02G193300.1	1	V	1680	2B	reverse	168831609	168853380	801256715	10	11	559	62.28	8.61	45.14	85.64	−0.230	secreted
*TaTPP6-A*	TraesCS2A02G412100.1	1	VI	1113	2A	forward	669749666	669753186	780798557	9	10	370	41.23	5.70	49.54	83.46	−0.307	chloroplast
*TaTPP6-B*	TraesCS2B02G430700.1	1	VI	1116	2B	forward	619679522	619682850	801256715	9	10	371	41.19	5.88	51.27	82.21	−0.297	chloroplast
*TaTPP6-D*	TraesCS2D02G409300.1	1	VI	1113	2D	forward	524105415	524108680	651852609	9	10	370	41.11	5.58	55.74	84.27	−0.232	chloroplast
*TaTPP7-A*	TraesCS3A02G085700.1	1	V	1662	3A	forward	55223622	55255982	750843639	11	12	553	61.55	8.06	36.82	83.67	−0.291	secreted
*TaTPP7-D*	TraesCS3D02G085800.1	1	V	1755	3D	forward	43259299	43283531	615552423	13	14	584	65.02	8.86	32.8	77.4	−0.400	mitochondrion
*TaTPP8-A*	TraesCS5A02G190000.1	1	I	1122	5A	reverse	394181080	394183400	709773743	5	6	373	40.85	8.95	41.99	79.81	−0.144	chloroplast
*TaTPP8-B*	TraesCS5B02G193100.1	3	I	1248	5B	reverse	348448002	348450302	713149757	4	5	373	40.93	8.97	41.35	79.81	−0.145	chloroplast
*TaTPP8-D*	TraesCS5D02G200800.1	1	I	1122	5D	reverse	303758772	303761166	566080677	5	6	373	40.86	8.96	42.04	79.81	−0.142	chloroplast
*TaTPP9-A*	TraesCS6A02G248400.1	1	VI	1119	6A	forward	461143866	461147635	618079260	8	9	372	41.11	5.68	48.98	85.65	−0.218	chloroplast
*TaTPP9-B*	TraesCS6B02G276300.1	1	VI	1119	6B	reverse	500209451	500213722	720988478	8	9	372	41.28	5.89	49.51	85.89	−0.232	chloroplast
*TaTPP9-D*	TraesCS6D02G230500.1	1	VI	1119	6D	forward	323712099	323716021	473592718	8	9	372	41.05	5.56	49.53	86.42	−0.192	chloroplast
*TaTPP10-A*	TraesCS6A02G301800.1	1	I	1251	6A	reverse	535151913	535154867	618079260	8	9	416	45.37	9.26	44.89	72.28	−0.305	other
*TaTPP10-B*	TraesCS6B02G330900.1	3	I	1224	6B	reverse	581079293	581082545	720988478	7	8	407	44.29	8.64	42.6	77.69	−0.147	mitochondrion
*TaTPP10-D*	TraesCS6D02G281100.1	1	I	1110	6D	reverse	388537685	388540648	473592718	8	9	369	40.25	8.79	37.81	76.99	−0.164	other
*TaTPP11-A*	TraesCS7A02G180800.1	1	I	1086	7A	reverse	135006112	135008690	736706236	9	10	361	39.54	8.41	50.69	81.39	−0.219	chloroplast
*TaTPP11-B*	TraesCS7B02G085800.1	1	I	1095	7B	reverse	97972425	97975038	750620385	9	10	364	40.16	8.11	50.16	79.09	−0.274	chloroplast
*TaTPP11-D*	TraesCS7D02G182600.1	1	I	1092	7D	reverse	136013159	136015620	638686055	9	10	363	39.91	8.60	51.06	79.56	−0.268	chloroplast

PC, Phylogenetic clade; ORF, Open Reading Frame; No, Number; bp, Base pair; Chr, Chromosome; aa, Amino Acid; M.W., Molecular Weight; Pi, Iso electric point; GRAVY, Grand average of hydropathy, SL, Subcellular Localization.

## Data Availability

Not applicable.

## Supplementary Materials

The following are available online at https://www.mdpi.com/article/10.3390/genes12111652/s1 Figure S1. Schematic illustration of 3-D protein structures and the Ramachandran plot for TaTPP proteins, Figure S2. Schematic representation of protein alignment of all TaTPPs, Figure S3. Schematic representation of TaTPP1-A side chains catalytic triads, Figure S4. Heat map of *TaTPPs* transcriptions in wheat development stages, Figure S5. Protein–protein interaction analysis of TaTPP proteins, Figure S6. Radiation tree of TaTPPs along with TPPs from other species, Table S1. List of primers used for *TaTPPs* qRT-PCR analysis, Table S2. TPP protein sequences identified from wheat genome, Table S3. Number of TPP proteins in different plant species, Table S4. Sequence identity and query cover of TaTPP proteins, Table S5. Pairwise identities and divergence between *TaTPP* genes and details about the duplication of those genes, Table S6. Phylogenetic tree member with their gene ID, Table S7. The synteny relationships of wheat *TPP* genes with different plant species, Table S8. Details of the calculated secondary structure elements TaTPPs by SOPMA, Table S9. Validation of TaTPP protein structures, Table S10. Frequency of all identified *Cis*-Regulatory Elements (CREs) in different *TaTPPs* promoters, Table S11. *Cis*-Regulatory Elements (CREs) with sequences and functions, Table S12: The protein-protein interaction network between TaTPPs and other proteins in wheat.
