# Supplementary material for: In Silico and Transcription Analysis of Trehalose-6-phosphate Phosphatase Gene Family of Wheat: Trehalose Synthesis Genes Contribute to Salinity, Drought Stress and Leaf Senescence
Source: Genes (Basel). 2021 Oct 20;12(11):1652. doi: 10.3390/genes12111652 (PMC8618227; doi:10.3390/genes12111652)
Supplement: Supplementary file 1 [file genes-12-01652-s001.zip › Supplementary/Supplementary Figure S1-6.pdf]

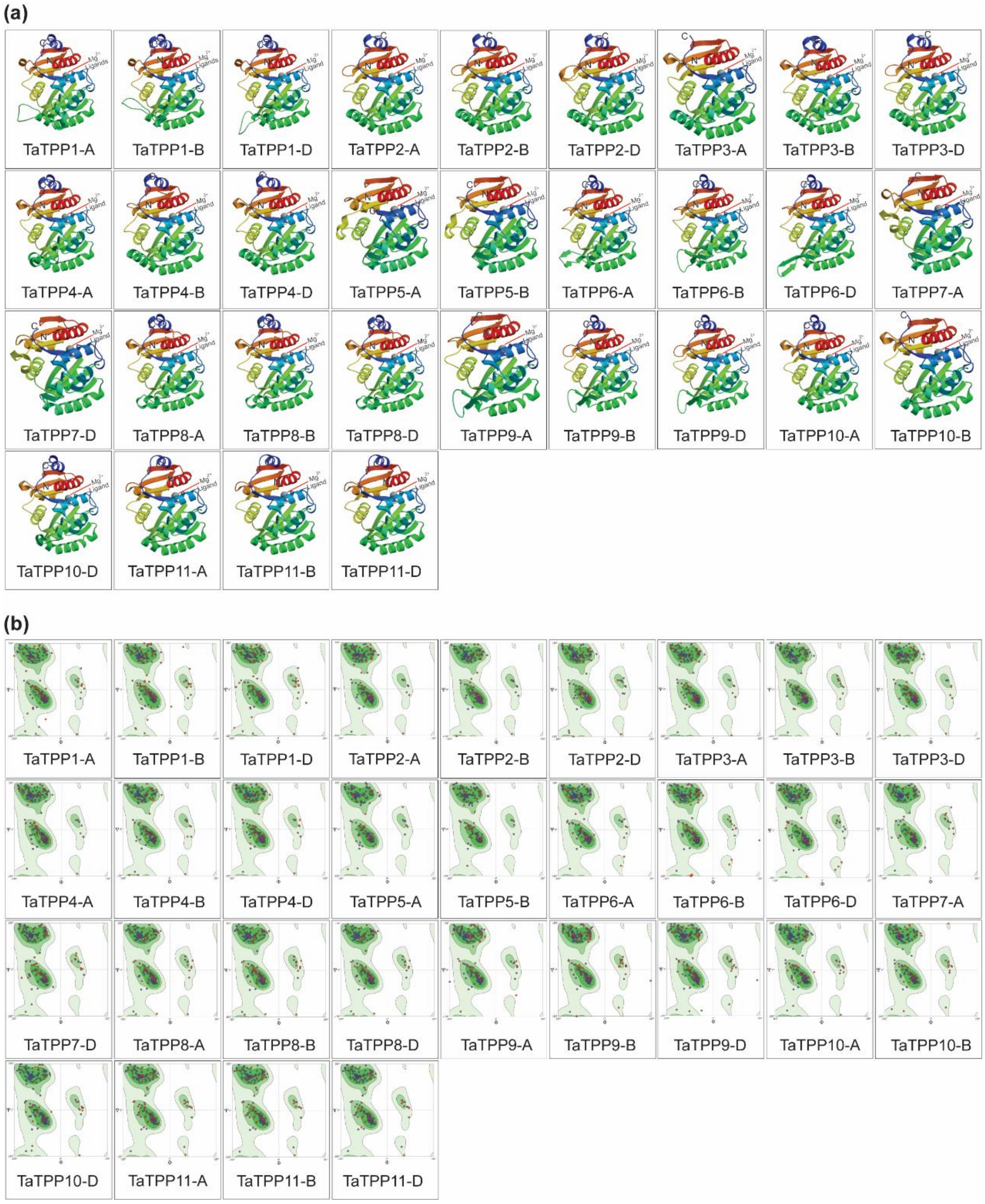

Figure S1. Schematic illustration of 3-D protein structures and the Ramachandran plot for TaTPP proteins. (a) TaTPP proteins predicted three-dimensional structures. N, N-terminus; Mg^{2+}, magnesium ion, C, C-terminus; (b) The Ramachandran plot for TaTPP proteins.

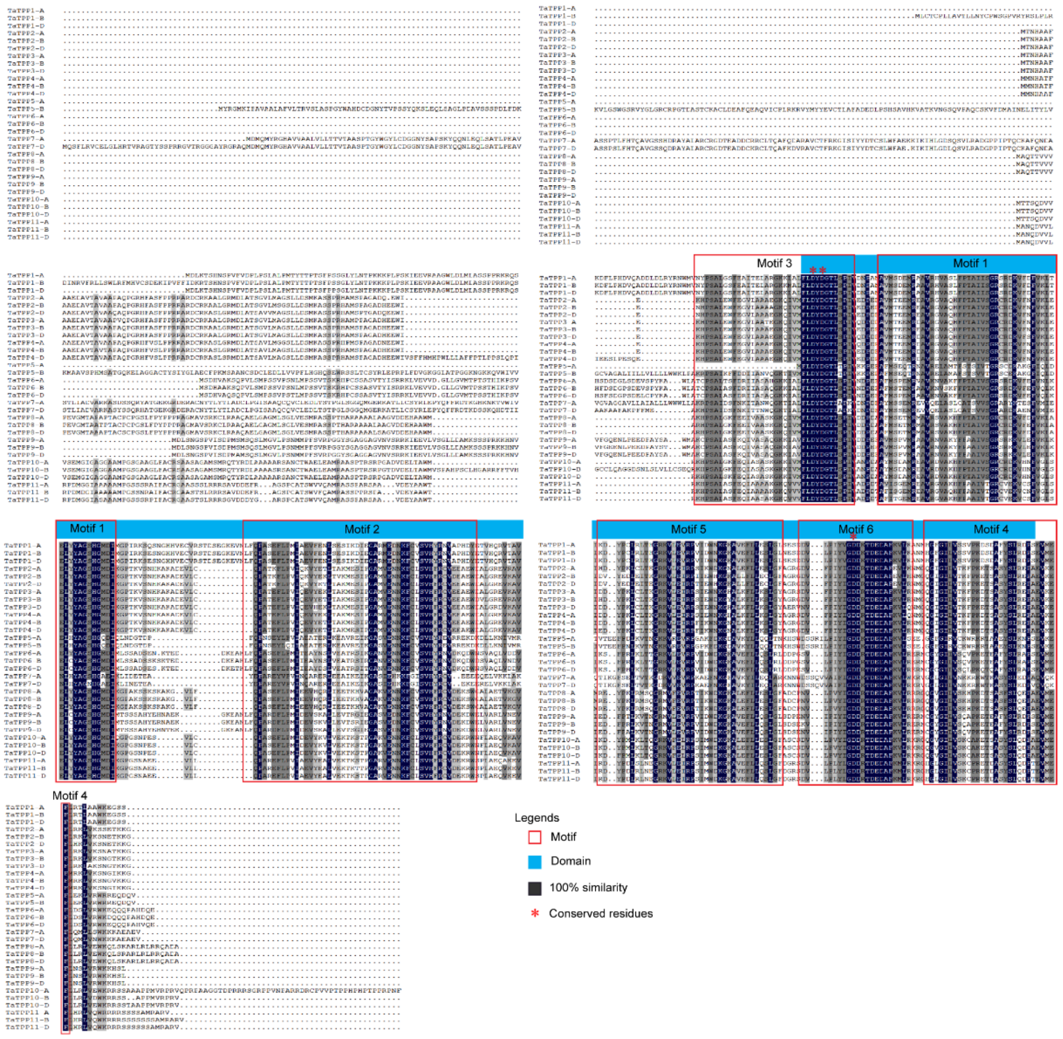

Figure S2. Schematic representation of protein alignment of all TaTPPs.

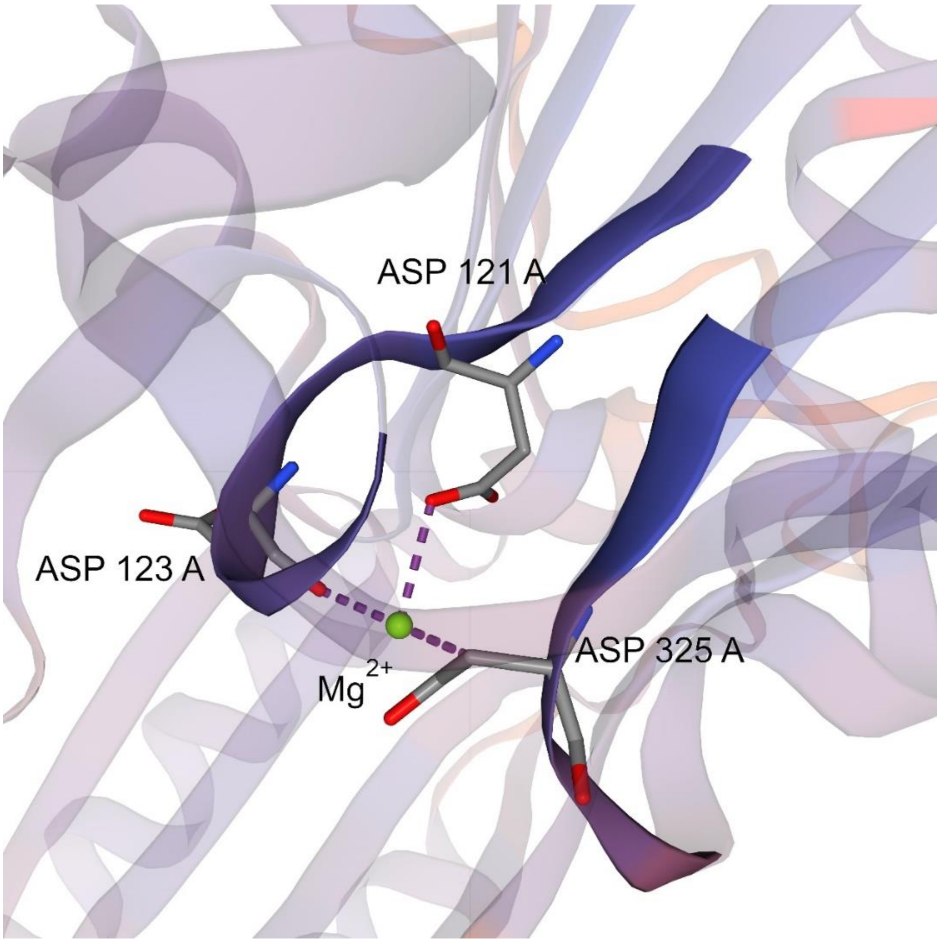

Figure S3. Schematic representation of TaTPP1-A side chains catalytic triads

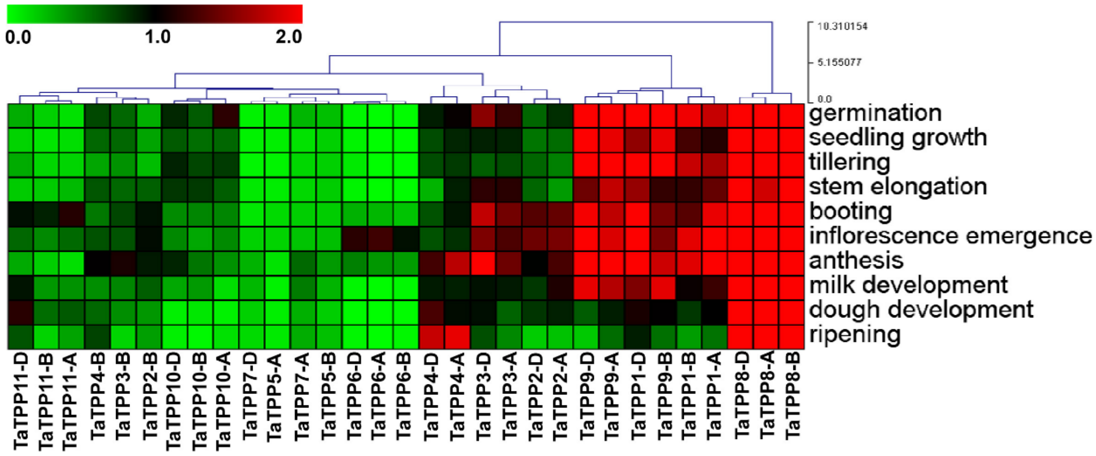

Figure S4. Heat map of *TaTPPs* transcriptions in wheat development stages. Gene transcriptions data was collected from Genevestigator.

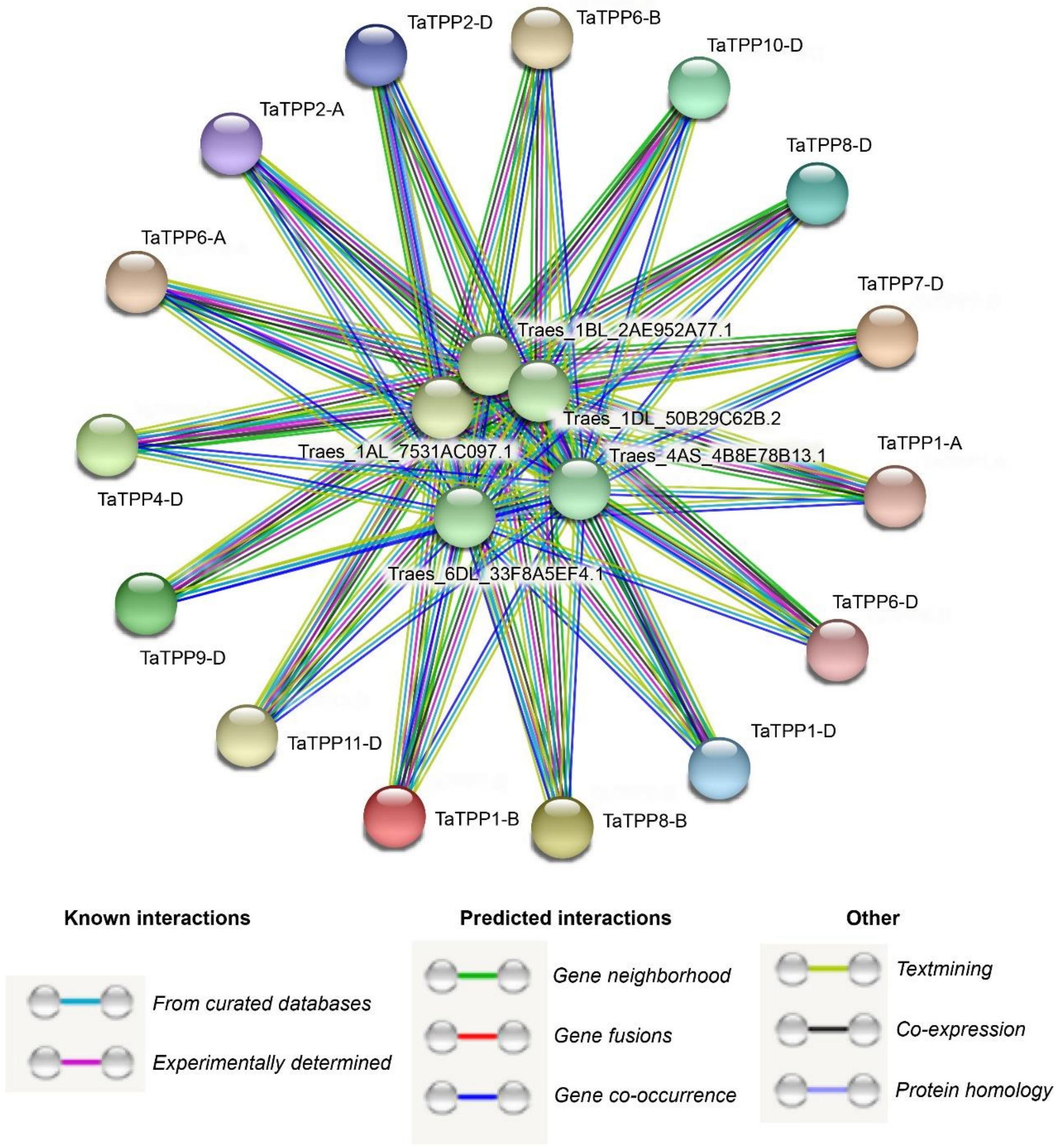

Figure S5. Protein–protein interaction analysis of TaTPP proteins. Protein–protein interaction network was produced by STRING V11.5, where each node represents a protein and each edge represents an interaction, colored by evidence type.

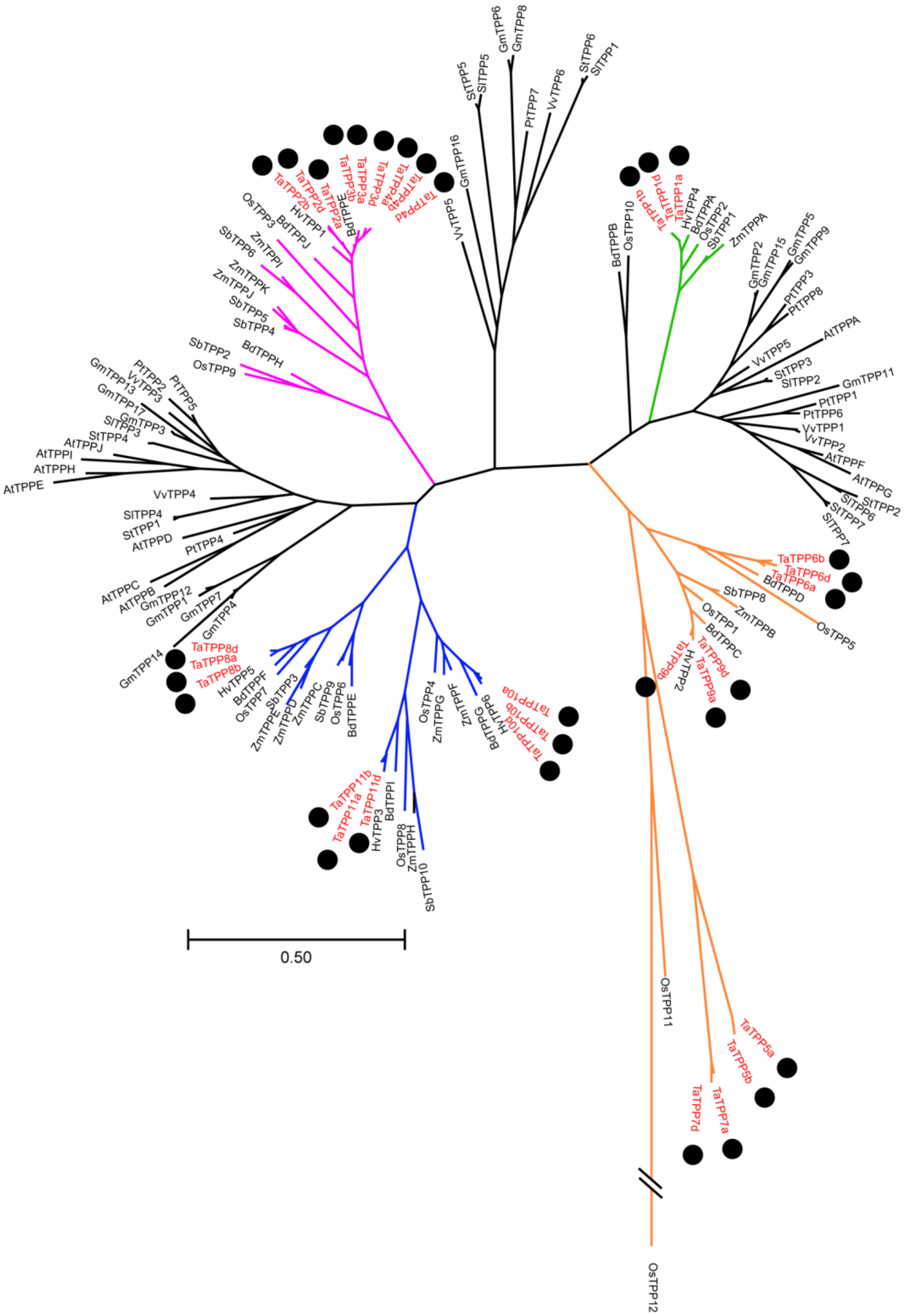

Figure S6. Radiation tree of TaTPPs along with TPPs from other species. *Os Oryza sativa* (Rice), *Zm Zea mays* (Maize), *Hv Hordeum vulgare* (Barley), *Sb Sorghum bicolor* (Sorghum), *Bd Brachypodium distachyon* (Purple false brome), *At Arabidopsis thaliana* (*Arabidopsis*), *Vv Vitis vinifera* (Grapevine), *Pt Populus trichocarpa* (Black cottonwood), *Gm Glycine max* (Soybean), *Sl Solanum lycopersicum* (Tomato), *St Solanum tuberosum* (Potato).
